# Digital Health Adoption Among Patients with Diabetes inQassim Unaizah, Saudi Arabia: A Cross-Sectional Study

**DOI:** 10.3390/healthcare14162654

**Published:** 2026-08-21

**Authors:** Nada Abdelrahman M. Ibrahim, Mohammed Saif Anaam, Talal Sami Alkeraidees, Bader Ayman Alsaegh, Mabrouk AL-Rasheedi, Majd Abdullah Alharbi, Ghaidaa Hamad Almotairi, Rama Mohammed Aldubaikhy, Waleed M. Altowayan

**Affiliations:** 1 Department of Pharmacy Practice, College of Pharmacy, Qassim University, Buraydah 51452, Qassim, Saudi Arabia; 2 College of Pharmacy, Qassim University, Buraydah 51452, Qassim, Saudi Arabiamajdabdullahal@outlook.com (M.A.A.);; 3 Researches and Studies Unit, Sukkar Association for Diabetes Education in Unaizah, Unaizah 51911, Qassim, Saudi Arabia; 4 Department of Dentistry, College of Dentistry, Qassim University, Buraydah 51452, Qassim, Saudi Arabia; 5 College of Dentistry and Pharmacy, Buraydah Colleges, Buraydah 51452, Qassim, Saudi Arabia; 6 Albukairyah General Hospital, Albukairyah 52725, Qassim, Saudi Arabia; 7 Department of Pharmaceutical Sciences, College of Pharmacy, Princess Nourah bint Abdulrahman University, Riyadh 11671, Riyadh, Saudi Arabia

**Keywords:** cross-sectional study, digital health, diabetes mellitus, telemedicine, questionnaire, Technology Acceptance Model, Saudi Arabia

## Abstract

**Highlights:**

**What are the main findings?**

- Digital health adoption in this cohort is remarkably high: nearly all participants owned smartphones (99.5%), over 80% used glucose tracking and medication reminder apps, and almost two-thirds used these tools daily. What really drove adoption was not how easy the tools were to use, but how useful patients found them (mean 4.29) and their overall positive attitude toward the technology (r = 0.450).
- Privacy concerns were surprisingly low (mean 1.70), yet they still showed a significant link with behavioural intention (r = 0.309). This suggests patients are aware of privacy issues but do not let them get in the way of using digital health tools. Interestingly, adoption intention did not differ by age, gender, education, income, or type of diabetes—meaning these tools appeal broadly across patient groups.

**What are the implications of the main findings?**

- Since perceived usefulness and attitude matter most, efforts to promote digital health should focus on showing patients how these tools improve their diabetes control and overall wellbeing. While ease of use was a significant correlate of adoption, it was not the strongest driver in this digitally literate cohort. However, usability and accessibility remain critically important, particularly for populations with lower digital literacy or limited technology experience. Technology developers should continue to prioritize intuitive design while also demonstrating clinical value.
- The consistency of adoption intentions across demographic subgroups in this cohort suggests that digital health solutions may have broad appeal across diverse patient populations in similar settings. However, our convenience sample from a single city limits generalizability, and findings should be confirmed in larger, more diverse samples—particularly among older adults and those with lower digital literacy. The low uptake of advanced tools like CGM (8.8%) and video consultations (1.0%) highlights the need for further investigation into the barriers to their use—including clinical appropriateness, cost, reimbursement, availability, and patient awareness—to ensure these technologies are accessible to those who could benefit most.

**Abstract:**

Background: Diabetes mellitus is a major public health concern in Saudi Arabia, affecting approximately 18.3% of adults. Although over 95% of people in the Qassim region own smartphones, limited information exists regarding how and why patients with diabetes utilize digital health technologies. Objective: This study employed an extended Technology Acceptance Model (TAM) incorporating trust, privacy concerns, and self-efficacy to investigate the factors influencing digital health technology adoption among patients with diabetes in Qassim Unaizah, Saudi Arabia. Methods: A cross-sectional study was conducted from November 2025 to January 2026 following institutional review board approval. The quantitative phase utilized a structured survey (n = 203) measuring TAM constructs via validated 5-point Likert scales. Descriptive statistics, Pearson correlations, and one-way ANOVA were performed. Qualitative themes were derived from open ended responses to contextualize quantitative findings. Results: Most participants were male (62.6%), with a mean age of 47.2 years (SD ± 13.1). Smartphone ownership was nearly universal (99.5%), and 82.8% used blood glucose tracking applications. All TAM constructs exhibited significant positive correlations with behavioural intention (*p* < 0.001): attitude (r = 0.450), perceived usefulness (r = 0.438), self-efficacy (r = 0.394), trust (r = 0.379), ease of use (r = 0.340), and privacy concern (r = 0.309). Mean scores indicated strong acceptance: perceived usefulness (4.31, SD ± 0.39), behavioural intention (4.21, SD ± 0.44), and attitude (4.17, SD ± 0.42). Daily usage was reported by 63.1% of participants, 81.8% expressed satisfaction, and 63.1% reported that digital tools greatly improved their diabetes management. Privacy concerns were notably low (mean 1.70, SD ± 0.89, reverse-coded; Cronbach’s α = 0.921). Supplementary qualitative content analysis of optional open-ended comments (n = 40 respondents) identified six recurring topics: clinical utility, digital literacy, trust, data security awareness, patient empowerment, and social support. Conclusions: Patients with diabetes in Qassim Unaizah demonstrate substantial digital health adoption, predominantly driven by perceived usefulness, positive attitudes, and self-efficacy. The extended TAM effectively explains adoption within this Saudi Arabian context. Findings support interventions emphasizing clinical benefits, user friendly design, and trust building to optimize digital health utilization in diabetes care.

## 1. Introduction

Diabetes mellitus has emerged as one of the most pressing public health challenges of the twenty-first century. Globally, approximately 589 million adults were living with diabetes in 2021, a number projected to rise to 853 million by 2050 [1]. The Kingdom of Saudi Arabia bears a particularly heavy burden, with prevalence rates reaching approximately 18.3% among adults, placing the nation among the top ten countries worldwide for diabetes diagnosis [2,3]. Within the Qassim region, where Unaizah is located, the situation mirrors these national trends, with rising incidence rates placing increasing strain on an already overburdened healthcare system. The economic toll is substantial; diabetes-related healthcare expenditures in Saudi Arabia were estimated at 1.5 billion dollars in 2010, with projections suggesting costs could exceed 6.5 billion dollars annually as prevalence continues its upward trajectory [3].

The management of diabetes poses unique challenges for both patients and clinicians. Unlike acute conditions that respond to episodic treatment, diabetes demands continuous, vigilant self-management encompassing dietary monitoring, physical activity, medication adherence, and regular blood glucose testing [4]. For patients to successfully navigate these daily demands, they require not only medical knowledge but also high levels of health literacy, numeracy, and sustained motivation [5]. Yet even with optimal clinical guidance, many patients struggle to maintain consistent self-care routines, leading to suboptimal glycemic control and increased risk of debilitating complications [6].

In recent years, digital health technologies have emerged as a promising avenue for supporting diabetes self-management. Telemedicine applications, telemedicine platforms, continuous glucose monitoring (CGM) systems, and electronic patient portals offer unprecedented opportunities to extend clinical care beyond the traditional office visit [7].

These tools can provide real-time feedback, deliver educational content, facilitate remote communication with providers, and enable patients to track their own physiological data conveniently from home [4]. A growing body of evidence supports their effectiveness; meta-analyses have demonstrated that mobile phone-based interventions can reduce Glycated haemoglobin (HbA1c) levels by 0.3% to 0.8%, with particularly pronounced benefits for patients with type 2 diabetes [8]. Similarly, systematic reviews have shown that technology-enabled diabetes self-management solutions significantly improve clinical outcomes when they incorporate complete feedback loops involving two-way communication, patient-generated health data analysis, tailored education, and individualized feedback [7].

Saudi Arabia has made substantial investments in digital health infrastructure as part of its ambitious Vision 2030 initiative. The Ministry of Health has actively promoted digital health solutions, developing platforms such as Sehhaty, Tawakkalna, Mawid, and Wasfaty to transform healthcare delivery across the Kingdom [9]. With over 24 million users—approximately 68.5% of the population—the Sehhaty app has become integral to the nation’s digital health transformation, enabling appointment scheduling, electronic health record access, and remote communication with healthcare providers [10]. Smartphone penetration in the country exceeds 90%, and over 95% of the population has internet access [3,11]. The COVID-19 pandemic further accelerated adoption, as both patients and providers turned to digital solutions to maintain continuity of care during lockdowns and social distancing measures [9].

Despite this favorable technological landscape, adoption of digital health tools among patients with diabetes in Saudi Arabia remains incompletely understood. Research has shown that approximately 78% of Saudi patients with diabetes use eHealth resources, with Google and social media platforms being the most common sources [12]. However, concerning patterns have emerged: eHealth dependency—defined as replacing physician consultation with online information—has been associated with significantly lower diabetes self-management scores, particularly in healthcare utilization and glucose management [12]. Furthermore, studies have documented poor compliance with diet control and physical activity recommendations among Saudi patients with diabetes, suggesting that simply providing access to digital tools does not guarantee effective engagement [13]. These findings underscore the critical need to understand not merely whether patients use digital health technologies, but why they adopt—or fail to adopt—these tools, and how usage patterns relate to meaningful self-management behaviors.

TAM, originally developed by Davis (1989) [14], provides a robust theoretical framework for investigating these questions. TAM posits that perceived usefulness—the degree to which a person believes that using a system will enhance their performance—and perceived ease of use—the degree to which using the system is believed to be free of effort—are fundamental determinants of technology adoption intentions and subsequent behavior [14,15]. The model has been extensively validated across diverse information technology contexts and has demonstrated particular utility in healthcare settings [16]. However, researchers have increasingly recognized that the original TAM may not fully capture the complexities of health technology adoption, where concerns about data privacy, trust in the technology and its providers, and patients’ confidence in their own abilities to use digital tools (self-efficacy) play important mediating roles [3,17]. Consequently, extended TAM frameworks incorporating these additional constructs have shown improved explanatory power for health-related technology acceptance.

The present study was therefore designed to address several critical gaps in the existing literature. First, while TAM has been extensively studied in Western and East Asian contexts, its applicability to Middle Eastern healthcare environments—particularly Saudi Arabia—remains underexplored [18]. Cultural factors, including attitudes toward technology, privacy expectations, and trust in digital health systems, may substantially influence adoption patterns in ways that differ from populations studied previously. Second, the majority of existing research has employed either purely quantitative survey designs or purely qualitative interview approaches, with relatively few studies integrating both methodologies to provide a comprehensive understanding of adoption phenomena [19]. Third, localized data from the Qassim region, which has unique demographic and healthcare delivery characteristics, have been notably absent from the national discourse on digital health adoption.

To address these gaps, we conducted a cross-sectional study using an extended TAM framework that incorporated trust, privacy concerns, and self-efficacy alongside the traditional TAM constructs. Our primary objective was to identify the factors influencing digital health technology adoption among patients with diabetes in Qassim Unaizah, Saudi Arabia. Secondary objectives included evaluating the prevalence of digital health tool utilization, identifying specific obstacles and enablers of adoption, analyzing correlations between patient characteristics and adoption behaviors, and formulating evidence-based recommendations to enhance digital health integration into routine diabetes care. We hypothesized that perceived usefulness, ease of use, attitude, trust, self-efficacy, and privacy concerns would each demonstrate significant associations with behavioral intention to use digital health technologies, consistent with extended TAM predictions. By employing a cross-sectional design with supplementary qualitative content analysis, we sought not only to quantify these relationships but also to contextualize the quantitative findings with insights from participant comments.

## 2. Materials and Methods

### 2.1. Study Design and Rationale

This study employed a cross-sectional survey design with supplementary qualitative content analysis. This approach allowed us to collect quantitative data and qualitative insights concurrently, then bring them together to gain a fuller picture of why and how patients with diabetes in Qassim, Unaizah use digital health technologies. The quantitative component provided numerical data on usage patterns and patient perceptions, while the supplementary qualitative component—derived from optional free-text comments—helped contextualize and illustrate the quantitative findings. We refer to this as supplementary rather than a full mixed-methods design because the qualitative data were limited to brief comments rather than in-depth interviews, and the analysis was intended to complement rather than triangulate the quantitative findings.

### 2.2. Setting and Participants

The study took place in Unaizah, a city in the Qassim region of Saudi Arabia. We recruited adult patients with diabetes from primary healthcare centres and a secondary care diabetes clinic. To be included, participants had to (1) have a confirmed diagnosis of diabetes (Type 1 or Type 2), (2) be aged 14 years or older, (3) live in the Qassim region, and (4) be able to give informed consent (or, for minors aged 14–17, have parental or guardian consent and provide personal assent). We excluded people with severe cognitive problems who could not complete the survey on their own.

### 2.3. Sampling and Sample Size

We used convenience sampling, inviting every eligible patient who came to the clinics during the study period. A total of 218 patients were assessed for eligibility. Of these, 7 were excluded (5 did not meet the age criteria, and 2 had severe cognitive impairment preventing survey completion), leaving 211 eligible patients. All 211 eligible patients were approached and invited to participate. Eight patients declined, citing lack of interest, time constraints, or feeling unwell. The remaining 203 patients agreed to participate and started the survey; all 203 completed it in full, yielding a completion rate of 100% among those who started the survey. To ensure our sample was large enough for the planned statistical analyses, we performed a power calculation. Based on an expected correlation (r) of 0.20 between TAM constructs and behavioural intention, with alpha = 0.05 and power = 0.80, we needed at least 193 participants. We ultimately recruited 203 participants, exceeding the required sample size.

### 2.4. Data Collection

Data were collected between November 2025 and January 2026. We used an online survey built with Google Forms. Trained research assistants approached patients in the waiting rooms of the participating clinics, explained the study, and gave them a tablet or a QR code to access the survey. Patients who preferred a paper version were given a printed copy. Most people (about 85%) used the online version, which took between 10 and 15 min to complete. All responses were anonymous, and no identifying information was collected.

### 2.5. Survey Instrument

The questionnaire was developed in Arabic to make sure it was culturally appropriate and easy to understand. We adapted items from validated scales used in previous TAM studies [3,14] and modified the wording slightly to fit the local context. Translation was performed using a forward and backward translation method by a bilingual clinical pharmacist (native Arabic speaker), then back-translated independently by a second bilingual researcher; discrepancies were resolved by consensus. To ensure content validity, a panel of five clinical experts (three diabetes physicians, two clinical pharmacists) reviewed each item for relevance, wording clarity, and construct representativeness. Panel feedback resulted in the rewording of four items and the addition of one item clarifying the definition of “digital health tools. The survey had four main sections.

The first section collected sociodemographic and clinical information: age, gender, education level, employment status, monthly household income, marital status, type of diabetes (Type 1 or Type 2), duration of diabetes, current treatment (diet/exercise alone, oral medication, insulin, or combinations), most recent HbA1c level (if known), and whether the person had any diabetes-related complications.

The second section asked about technology access and digital literacy: whether the participant owned a smartphone, how they accessed the internet (home, work, school, etc.), and how they would rate their own digital skills (poor, fair, good, excellent).

The third section focused on actual use of digital health tools. Participants could select from a list of common tools: blood glucose tracking apps, medication reminder apps, diet/nutrition apps, physical activity apps, continuous glucose monitoring systems, social media support groups, wearable devices, online diabetes education, video consultation platforms, and patient portals. They also reported how often they used these tools (daily, several times per week, weekly, less often, or never). For satisfaction and perceived improvement, all participants were asked these questions with a ‘not applicable’ option available; participants who reported never using digital health tools selected ‘not applicable’ and were excluded from the satisfaction and improvement calculations.

The fourth section measured the TAM constructs using 5-point Likert scales (1 = strongly disagree, 5 = strongly agree). We included the following scales, each with three or four items: perceived usefulness (PU, 3 items), perceived ease of use (PEOU, 4 items), attitude toward use (ATT, 3 items), behavioural intention (BI, 2 items), trust (TR, 2 items), privacy concern (PC, 3 items—reverse-coded, meaning a higher score indicates less concern), and self-efficacy (SE, 3 items).

The whole questionnaire was pilot tested with 15 patients with diabetes who were not part of the main study. Based on their feedback, we reworded a couple of items that sounded confusing and adjusted the order of some questions to make the flow more natural.

### 2.6. Quantitative Data Analysis

All statistical analyses were performed using SPSS version 26.0 (IBM, Armonk, NY, USA). First, we ran descriptive statistics: means and standard deviations for continuous variables, and frequencies with percentages for categorical variables. To assess the internal consistency of each TAM scale, we calculated Cronbach’s alpha. A value of 0.70 or higher is generally considered acceptable; however, we retained some scales with lower alpha coefficients because they consisted of few items and the constructs still made theoretical sense in our context.

Prior to analysis, we assessed the underlying assumptions: normality was evaluated using the Shapiro–Wilk test and Q–Q plots, homogeneity of variance for *t*-tests and ANOVA was examined using Levene’s test, and linearity for correlations was confirmed via scatterplots. No major violations of these assumptions were detected. For group comparisons, we conducted one-way ANOVA to determine whether BI differed by education level, age, or income. An independent-samples *t*-test was used to compare BI between male and female participants, as well as between type 1 and type 2 diabetes patients. All tests were two-tailed, and we considered a *p*-value less than 0.05 to be statistically significant. No missing data were present, as all participants who started the survey completed it in full. We used Pearson correlations coefficients to examine the direction and strength of bivariate associations between each TAM construct and behavioural intention, as well as the intercorrelations among the constructs themselves. We did not perform multivariable regression due to high intercorrelations among constructs (e.g., PU and PEOU correlated at r = 0.67) and the exploratory nature of this study.

### 2.7. Qualitative Analysis

Although we did not conduct separate interviews, we derived supplementary qualitative insights from optional open-ended comments. At the end of the survey, participants were invited to provide additional written comments about their experiences with digital health tools. A total of 40 participants (19.7% of the sample) provided comments. The comments addressed various aspects of digital health tool use, including ease of use, clinical utility, trust, and suggestions for improvement.

Two researchers independently read through all comments and coded them using an inductive approach inspired by Braun and Clarke’s thematic analysis framework [20]. The researchers first familiarized themselves with the data, then generated initial codes independently, and subsequently met to discuss and refine codes. Coding was descriptive and focused on recurring topics mentioned by participants. For example, comments mentioning ‘helpful for tracking’ or ‘improved my control’ were coded under ‘clinical utility’; comments expressing confidence in app accuracy were coded under ‘trust’; and comments mentioning family, friends, or online groups were coded under ‘social support.’ Disagreements in coding were resolved through discussion until consensus was reached.

Through this process, we identified six recurring topics that captured the most important aspects of patients’ experiences: clinical utility, digital literacy, trust, data security awareness, patient empowerment, and social support. We present these as supplementary qualitative insights rather than formal qualitative themes, given the limited depth of the comment data. These insights are illustrated with representative quotations (with minor editing for clarity) and are used to contextualize and enrich the quantitative findings.

## 3. Results

### 3.1. Participant Characteristics

**Figure 1:**
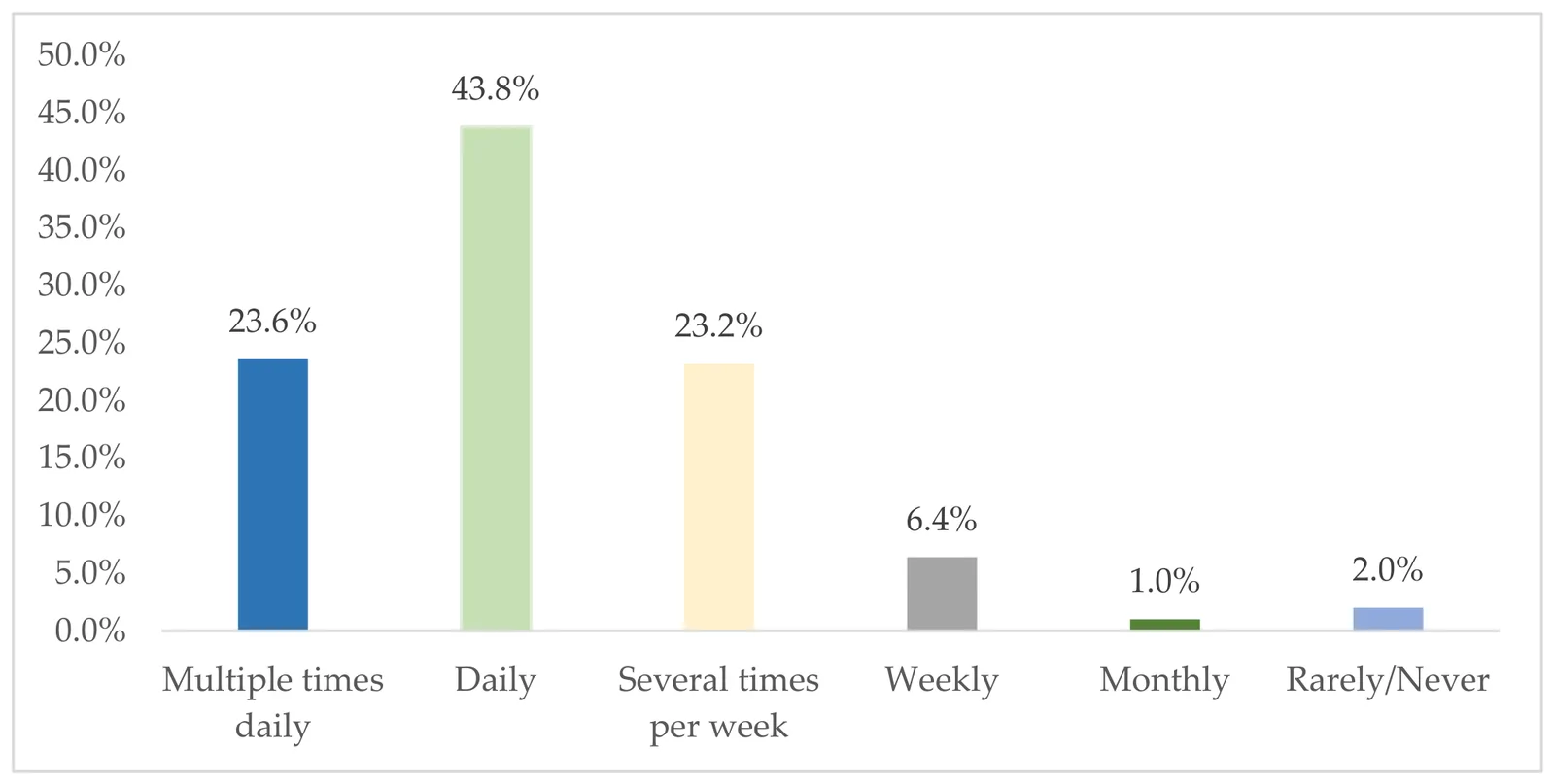
How often to check blood glucose.

A total of 203 patients with diabetes completed the survey, giving a response rate of 100% of those we approached. The average age was 47.2 years (SD 13.1), ranging from 14 to 82 years. The sample was predominantly male (62.6%, n = 127), with females comprising 37.4% (n = 76). More than half of the participants held a bachelor’s degree (55.7%, n = 113), followed by high school education (n = 42, 20.7%). Most were employed full time (58.6%, n = 119) or retired (20.2%, n = 41). Monthly household income was most commonly in the 10,000–14,999 SAR range (31.0%, n = 63) or 5000–9999 SAR (29.1%, n = 59). The majority were married (61.6%, n = 125). Table 1 summarises these demographic data, and Figure 1 provides a visual breakdown of key characteristics.

### 3.2. Clinical Characteristics

Type 2 diabetes was the most common diagnosis, affecting 61.6% (n = 125) of participants, followed by Type 1 diabetes (38.4%, n = 78). The majority of the participants (n = 77, 37.9%), had diabetes for 8–10 years. Regarding treatment, the largest group used a combination of diet/exercise plus oral medication (31.5%, n = 64). Oral medication alone was used by 26.1% (n = 53), and insulin injections by 20.2% (n = 41). HbA1c levels were predominantly in the 7.6–8.5% range (37.4%, n = 76), with 32.0% (n = 65) achieving target levels of 6.5–7.5%. A significant number of participants (n = 47, 23.2%), had a level of 8.6–9.5%. Thirty-eight (18.7%) participants reported diabetes-related complications (diabetes foot (n = 13), neuropathy (n = 11), retinopathy (n = 10); the remaining (n = 4) did not specified any complication). In regard to checking blood glucose, most of the participants (n = 89, 43.8%) check their blood glucose daily; Figure 1 shows more details.

### 3.3. Technology Access and Digital Literacy

Almost everyone owned a smartphone (99.5%, n = 202). Internet access was available at both home and work or school for 74.9% (n = 152), while another 16.7% (n = 34) had access only at home. Self-rated digital literacy was high: 69.5% (n = 141) described their skills as “excellent”, 22.2% (n = 45) as “good”, and only 2.0% (n = 4) as “poor”.

### 3.4. Use of Digital Health Tools

**Figure 2:**
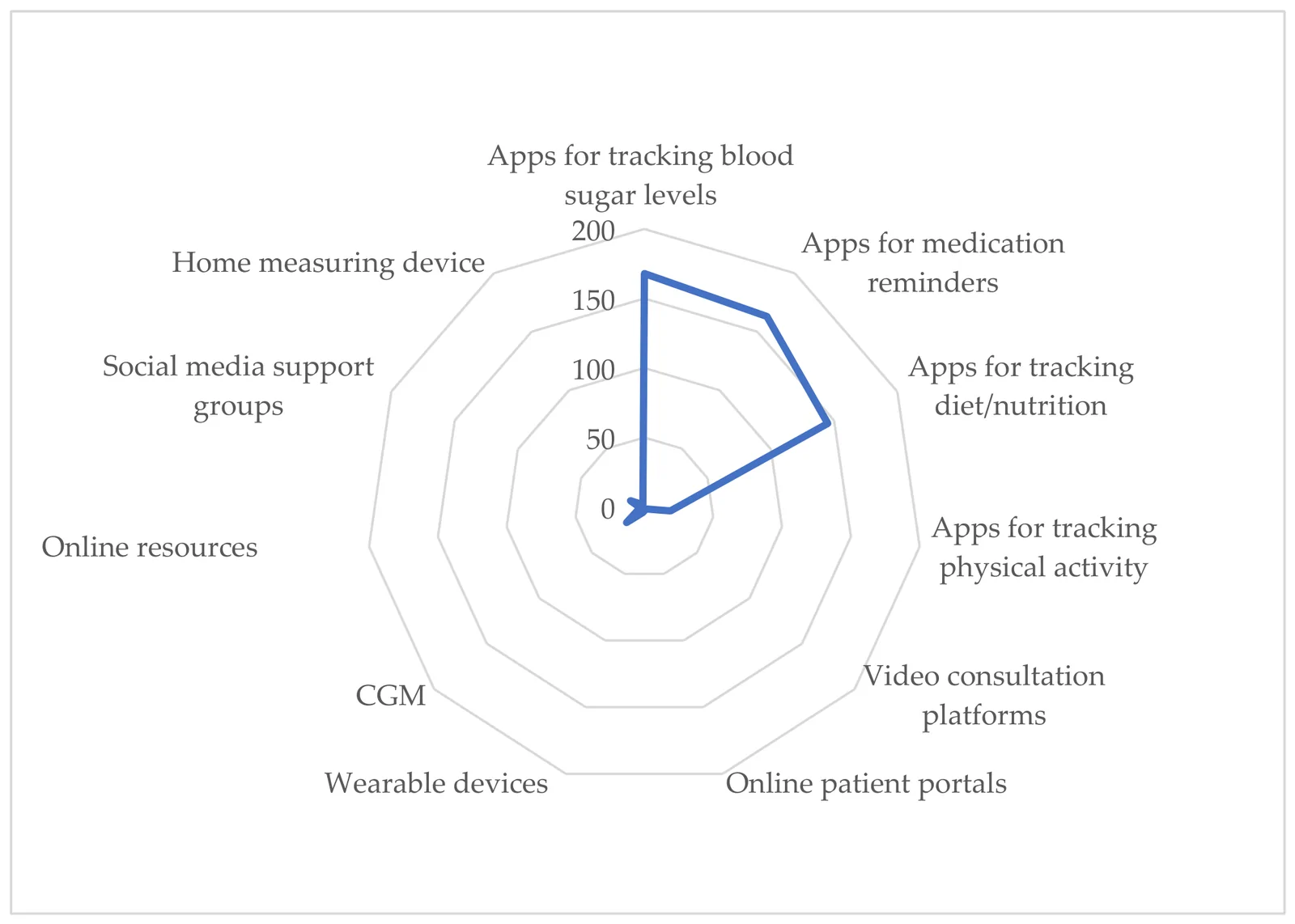
Digital health tools used in previous 6 months.

In the six months before the survey, blood glucose tracking applications were the most widely used digital health tool (82.8%, n = 168). Medication reminder applications were also very common (80.3%, n = 163), followed by diet and nutrition tracking apps (71.4%, n = 145). Physical activity apps were used by 9.4% (n = 19), and CGM by 8.8% (n = 17). Social media support groups (5.4%, n = 11), wearable devices (1.9%, n = 4), online diabetes education resources (1.9%, n = 4), video consultation platforms (1.0%, n = 2), patient portals (1.0%, n = 2), and home measuring devices (1.0%, n = 2) had much lower uptake (Figure 2).

Daily use of these tools was reported by 63.1% (n = 128), and another 26.6% (n = 54) used them several times per week. Monthly use was reported by 4.4% (n = 9). Only 5.9% (n = 11) reported that they never or rarely used digital health tools. For satisfaction and perceived improvement, all 203 participants responded to these questions; the 11 non-users (5.9%) selected the ‘not applicable’ option. Thus, using the entire sample (N = 203) as the denominator, overall satisfaction was high: 42.9% (n = 87) were ‘very satisfied’ and 38.9% (n = 79) were ‘satisfied’, giving a combined satisfaction rate of 81.8% (n = 157). Only 3.4% (n = 7) expressed dissatisfaction. A majority (63.1%, n = 128) said that digital health tools had ‘greatly improved’ their diabetes management. Among users (n = 192), the satisfaction rate was 81.8% (157/192) and the greatly improved rate was 66.7% (128/192).

### 3.5. Technology Acceptance Model Constructs

All TAM constructs, except for Privacy Concern had mean scores above the neutral midpoint of 3.0 on the 5-point scale. Perceived usefulness scored highest (mean 4.29, SD 0.39), followed by behavioural intention (mean 4.16, SD ± 0.44), attitude (mean 4.17, SD ± 0.42), trust (mean 4.22, SD ± 0.51), and ease of use (mean 4.11, SD ± 0.66). Self-efficacy showed a moderate to high score (mean 3.86, SD ± 0.53). Privacy concern had a low mean (1.70, SD ± 0.89, reverse-coded, where lower scores indicate greater concern), indicating that participants were generally concerned about privacy issues. Internal consistency, as measured by Cronbach’s alpha, varied across constructs (Table 2). PC (α = 0.921) and PEOU (α = 0.839) demonstrated excellent reliability, TR (α = 0.653) and BI (α = 0.498) showed moderate reliability, while PU (α = 0.378), ATT (α = 0.354), and SE (α = 0.316) fell below acceptable thresholds, which we discuss later as a limitation.

### 3.6. Correlation Analysis

All TAM constructs were significantly and positively correlated with BI (all *p* < 0.001). Attitude had the strongest correlation (r = 0.450), followed closely by PU (r = 0.438). SE (r = 0.394), TR (r = 0.379), PEOU (r = 0.340), and PC (r = 0.309) also showed significant, albeit somewhat weaker, associations (Table 3). As expected from TAM theory, PU and PEOU were themselves strongly correlated (r = 0.67). The highest inter-construct correlation was between Attitude and SE (r = 0.52).

### 3.7. Group Comparisons

A one-way ANOVA revealed no significant differences in BI scores based on education level (F = 1.09, *p* = 0.362), age (F = 0.95, *p* = 0.473), or income (F = 0.31, *p* = 0.909). Similarly, an independent-samples *t*-test showed no significant gender difference (t = −1.30, *p* = 0.196), although females had a slightly higher mean score (4.24) than males (4.12). Another independent-samples *t*-test also found no significant difference in scores by type of diabetes (t = −0.917, *p* = 0.360). Taken together, these findings suggest that BI was broadly similar across demographic and clinical subgroups in this sample. The mean BI scores across all subgroups fell within a narrow range (4.11–4.24), with no consistent pattern suggesting meaningful differences. While nonsignificance does not establish equivalence, the homogeneity of point estimates provides descriptive evidence of broad acceptance across these demographic categories within this cohort.

### 3.8. Supplementary Qualitative Insights

To contextualize the quantitative findings, we analyzed optional open-ended comments provided by 40 participants (19.7% of the sample). Six recurring topics were identified through inductive coding. Below, we describe each topic with representative quotations and indicate whether it was derived from comments, Likert patterns, or both.

1. Topic 1: Clinical Utility

Source: Primarily from comments; supported by high PU score (mean 4.29) and high usage of glucose tracking (82.8%) and medication reminder apps (80.3%).

Participants frequently mentioned the practical benefits of digital health tools for managing their diabetes. Comments emphasized how these tools helped with monitoring blood glucose and remembering medications.

Representative quotation: “The app helps me track my sugar levels daily; I can see my progress and share it with my doctor.”

(Participant 23, male, age 65)

Representative quotation: “I never forget my medication now because of the reminder.”

(Participant 104, female, age 57)

2. Topic 2: Digital Literacy and Ease of Navigation

Source: Primarily from Likert patterns (PEOU mean 4.11; 69.5% rated skills as excellent); supported by comments about ease of use.

Comments suggested that most participants found the tools easy to navigate, though some mentioned initial challenges.

Representative quotation: “At first, it was difficult, but after a few days it became very easy.”

(Participant 156, male, age 48)

Representative quotation: “I found the app very simple to use, even for someone like me who is not very tech-savvy.”

(Participant 187, female, age 44)

3. Topic 3: Trust in Digital Health Systems

Source: Primarily from Likert patterns (Trust mean 4.22; r = 0.379 with BI); comments expressed confidence in government platforms.

Several participants expressed trust in the accuracy and reliability of digital health tools, particularly government-endorsed platforms.

Representative quotation: “I trust the Sehhaty app because it’s from the Ministry of Health; I know my data is safe.”

(Participant 45, male, age 43)

4. Topic 4: Data Privacy and Security Awareness

Source: Primarily from Likert patterns (low PC mean 1.70); comments rarely mentioned privacy concerns directly, though some participants expressed a general preference for privacy.

Very few comments addressed data privacy directly, consistent with the low privacy concern score. However, some participants mentioned their data-sharing preferences.

Representative quotation: “I prefer not to share my data with everyone, only with my doctor.”

(Participant 78, male, age 40)

5. Topic 5: Patient Empowerment and Self-Management

Source: Primarily from Likert patterns (63.1% reported ‘greatly improved’ diabetes management; SE mean 3.86); comments reflected increased confidence.

Comments indicated that digital tools empowered participants to take greater control of their diabetes management.

Representative quotation: “The app made me more aware of my sugar levels and motivated me to eat better.”

(Participant 12, female, age 35)

Representative quotation: “I feel more in control of my diabetes since I started using the tracking app.”

(Participant 161, male, age 38)

6. Topic 6: Social and Community Support

Source: Primarily from comments; a small number of participants (5.4%) reported using social media support groups.

While few participants used social media support groups, those who did mentioned the value of peer support and shared experiences.

Representative quotation: “The WhatsApp group with other diabetic patients has been very helpful; we share tips and encourage each other.”

(Participant 91, female, age 33)

## 4. Discussion

This cross-sectional study with supplementary qualitative content analysis investigated the factors influencing digital health adoption among patients with diabetes in Qassim Unaizah, Saudi Arabia, using an extended TAM that incorporated trust, privacy concerns, and self-efficacy. Our main findings can be summarised as follows: (1) digital health tool adoption was high, with 82.8% of participants using blood glucose tracking apps and 80.3% using medication reminder apps; (2) perceived usefulness, attitude, self-efficacy, trust, ease of use, and privacy concerns were all significantly correlated with behavioural intention; (3) daily usage was reported by 63.1% of participants, and overall satisfaction reached 81.8%; (4) contrary to some international studies, privacy concerns were low; and (5) supplementary qualitative insights from optional open-ended comments highlighted recurring topics including clinical utility, digital literacy, trust, data security awareness, patient empowerment, and social support, which helped contextualize the quantitative findings.

### 4.1. Comparison with Existing Literature

Our findings align closely with the core propositions of the Technology Acceptance Model [14,15]. Perceived usefulness showed strongest correlation with behavioural intention (r = 0.438, *p* < 0.001), consistent with decades of TAM research in both general and healthcare information technology contexts. This is also in line with studies conducted in Saudi Arabia, where perceived usefulness emerged as a dominant driver of telemedicine adoption among patients with diabetes [21] and among the general population [3]. The high mean score for perceived usefulness (4.31 out of 5) suggests that patients in Qassim Unaizah genuinely believe that digital health tools enhance their ability to manage diabetes, a perception that likely explains the high daily usage rate (63.1%).

Attitude toward use showed the strongest correlation with behavioural intention (r = 0.450). This finding is somewhat at variance with the original TAM, where perceived usefulness often mediates the effect of attitude [14]. However, in healthcare settings where patients have strong personal motivations, attitude may play a more direct role. Similar results have been reported by Or and Karsh [17] in their systematic review of patient acceptance of consumer health information technology, where attitude was frequently a significant independent correlate.

Self-efficacy (r = 0.394) and trust (r = 0.379) also emerged as important correlates. The moderate to high self-efficacy score (mean 3.86) reflects the excellent digital literacy reported by participants (69.5% rating their skills as excellent). This is consistent with Bandura’s social cognitive theory, which posits that self-efficacy beliefs are foundational to health behaviour change [22]. In the diabetes self-management literature, autonomous motivation—a concept closely related to self-efficacy—has been shown to predict better adherence to diet and blood glucose testing [5]. The significant correlation between trust and behavioural intention underscores the importance of confidence in the accuracy, reliability, and security of digital health systems. However, our trust measure assessed general confidence in digital health tools rather than specific trust in government platforms, and we did not directly measure participants’ trust in the Ministry of Health or its platforms. This finding aligns with previous studies from Saudi Arabia, where trust was a key factor in patients’ willingness to use e-Health services [3].

One of the most striking findings was the low level of privacy concern (mean 1.70 on a 5-point scale, reverse-coded). This contrasts sharply with studies from Western countries, where privacy and security concerns consistently rank among the top barriers to telemedicine adoption [23,24]. Several speculative explanations, derived from the external literature, may account for this difference; however, we emphasize that these were not directly measured in our study and should be interpreted as hypotheses requiring future investigation. First, the Sehhaty app is a government-managed platform endorsed by the Saudi Ministry of Health, and government endorsement may confer a sense of security that mitigates privacy worries [10]. Second, cultural attitudes toward data sharing in Saudi Arabia may differ from those in Europe or North America; patients may place greater trust in institutional safeguards and have less individualistic concerns about data autonomy [18]. Third, the high digital literacy and frequent use of social media in the region may have normalised data sharing to some extent [11]. Nonetheless, the significant correlation (r = 0.309) between privacy concern and behavioural intention indicates that even low levels of concern can influence adoption decisions, a nuance that should not be overlooked. Future research should directly measure institutional trust, cultural attitudes toward data sharing, and knowledge of privacy risks to test these speculative explanations.

Our findings showed that 63.1% of participants used digital health tools daily and 81.8% were satisfied, exceeding the adoption rates reported in earlier Saudi studies. For example, Nasser et al. [12] found that 78.1% of Saudi patients with diabetes used eHealth resources, but only 44.2% were “eHealth dependent” (replacing physician consultation). The higher satisfaction in our sample may reflect the maturation of digital health platforms since the COVID-19 pandemic, which accelerated both availability and user familiarity [9]. It may also be due to the relatively high educational level of our sample (56.2% held a bachelor’s degree), which has been associated with greater technology acceptance [17].

In contrast to studies that reported age as a significant barrier [25,26], we found no significant effect of age on behavioural intention in our ANOVA results, with mean BI scores remaining consistent across age categories. Similarly, no significant differences were observed for gender, education, income, or diabetes type. These findings align with previous Saudi studies that found demographic characteristics to be weak or inconsistent predictors of digital health adoption [3,18,21], suggesting that attitudinal and contextual factors—particularly perceived usefulness and trust—may be more important targets for intervention than demographic tailoring. However, the mean age of our sample was 47.2 years, which is relatively young for a diabetic population in Saudi Arabia. Older patients (65+ years) were underrepresented, which may have attenuated age-related differences. Furthermore, nonsignificance does not establish equivalence; we interpret these findings as hypothesis-generating and emphasize the need for replication in larger, more diverse samples. Future research should specifically target older adults and those with lower digital literacy to better understand their unique barriers and facilitators.

### 4.2. Interpretation of Differences

How can we explain the divergence between our findings and those of studies reporting high privacy concern as a major barrier [27,28]? One possibility is that the Saudi government’s active role in digital health—through platforms like Sehhaty and the Seha Virtual Hospital—has normalised data sharing and built institutional trust. Another possibility is that participants in our study were less aware of potential privacy risks, a finding that would be concerning and warrants targeted patient education. The open-ended comments we analysed did not reveal active concerns about data breaches; instead, participants were more focused on technical performance (crashes, slow responsiveness) as noted by Alzghaibi [10]. This suggests that for this population, technical reliability may be a more immediate barrier than privacy.

Another difference concerns the role of ease of use. While ease of use was significantly correlated with behavioural intention (r = 0.340), its strength was lower than in many TAM studies [3,14]. This may be because our participants were highly digitally literate (69.5% rated their skills as excellent); for them, ease of use may be a baseline expectation rather than a distinguishing factor. However, we caution against interpreting this finding as justification for deprioritizing usability. In populations with lower digital literacy—including older adults, those with limited education, and individuals in rural areas—ease of use would likely be a stronger and more critical determinant of adoption. Moreover, the significant correlation we observed (r = 0.340, *p* < 0.001) confirms that usability remains a meaningful factor even in this digitally proficient sample. Technology developers should continue to prioritize intuitive, accessible design while also demonstrating clinical value to maximize adoption across all patient groups.

The low uptake of CGM (8.8%), telemedicine platforms (1.0%), and patient portals (1.0%) despite high smartphone ownership and internet access warrants careful interpretation. For CGM, uptake is appropriately influenced by several factors beyond patient preference, including diabetes type (CGM is primarily indicated for type 1 diabetes and insulin-treated type 2 diabetes), treatment regimen, clinical indication (e.g., hypoglycemia unawareness, pregnancy), cost, reimbursement policies, and device availability [29,30]. However, we did not directly assess these factors in our survey, and our data cannot determine whether low uptake reflects clinical appropriateness, lack of awareness, cost barriers, or other reasons. Not all patients with diabetes are candidates for or would benefit equally from CGM, and higher uptake is not inherently desirable without clinical appropriateness. The low video consultation uptake is consistent with national patterns; Almoajel et al. [9] similarly found that fitness tracking apps were far more commonly used than virtual consultation platforms. Several speculative explanations may account for this, derived from the external literature. First, patients may prefer face-to-face interaction for complex medical decisions, a finding reported in earlier telemedicine research [31]. Second, our participants were recruited from in-person clinics, and the survey did not assess whether they had a clinical need for or opportunity to use telemedicine services. Third, awareness of these services may remain low; only 20% of participants in a national survey reported that a doctor had recommended a health app [27]. Fourth, technical barriers (e.g., platform crashes, slow responsiveness) and usability issues have been identified as obstacles to mHealth utilization in Saudi Arabia [10]. Thus, rather than simply promoting higher uptake, future research should investigate: (a) clinical appropriateness and patient candidacy for these tools, (b) awareness and provider endorsement, (c) technical and usability barriers, and (d) cost, reimbursement, and infrastructure factors. Our data can only highlight low uptake; it cannot explain its underlying causes.

### 4.3. Strengths and Limitations

This study has several strengths. First, the use of an extended TAM framework that included trust, privacy, and self-efficacy provides a more nuanced understanding of adoption than the original model alone. Second, the mixed methods design allowed us to complement quantitative correlations with qualitative themes derived from participant comments, offering a richer interpretation. Third, the sample size (N = 203) exceeded the power calculation requirement, lending confidence to the statistical findings. Fourth, by focusing on a specific region (Qassim Unaizah) rather than a national sample, we provide localised evidence that can inform regional health policy.

Several limitations must be acknowledged. First, convenience sampling limits the generalisability of our findings. Our sample was relatively well educated and had high digital literacy, which may not represent all patients with diabetes in Qassim, particularly those with lower socioeconomic status or limited education. This recruitment bias has important implications for interpreting our findings regarding ease of use. The relatively weaker correlation observed for ease of use (r = 0.340) compared to perceived usefulness (r = 0.438) may be an artifact of our digitally proficient sample, for whom usability barriers are minimal. In less digitally literate populations, ease of use would likely emerge as a stronger and potentially dominant predictor of adoption. Therefore, our findings should not be interpreted as evidence that usability can be deprioritized in technology design or implementation strategies. While our sample’s high smartphone ownership (99.5%) aligns with regional estimates (>95%) and national penetration rates (>90%) [3,11], the overrepresentation of participants with bachelor’s degrees (55.7%) and excellent self-rated digital literacy (69.5%) may limit the transferability of our findings to less advantaged populations. Furthermore, while we found no significant differences in behavioral intention across demographic subgroups, we caution against interpreting this as definitive evidence of equivalence or universal appeal. Our sample may have limited power for subgroup analyses, particularly for smaller subgroups (e.g., participants aged 65+ years, n = 15; participants with primary education or less, n = 7). We present these subgroup findings as hypothesis-generating observations that warrant replication in larger, more diverse samples. Second, the cross-sectional design prevents causal inferences; we can only report associations, not direction of causality. Third, self-reported data are subject to recall bias and social desirability bias. Participants may have over reported their use of digital health tools or their satisfaction because they wanted to appear “technologically savvy”. Fourth, several Cronbach’s alpha values were below conventional thresholds (PU α = 0.378, ATT α = 0.354, BI α = 0.498, SE α = 0.316), indicating poor internal consistency. This may be attributed to the brevity of scales (2–3 items per construct), cultural adaptation of items, and restricted variance due to high mean scores. These findings should therefore be interpreted with caution, and future research should validate the measurement model using confirmatory factor analysis in this population. Additionally, the low alpha for behavioural intention (BI α = 0.498) indicates that this measure requires further validation. While the significant correlations observed are consistent with TAM predictions and theoretical expectations, these findings should be considered preliminary until replicated with more robust instruments. Fifth, our supplementary qualitative data were derived from optional open-ended survey comments provided by only 40 participants (19.7% of the sample), rather than in-depth interviews or focus groups. The comments were brief, which limited the depth of experiential insights. Consequently, we present these as recurring topics derived from brief comments, not as formal qualitative themes with the rigor of a full qualitative study. We did not achieve thematic saturation, and some topics were inferred from quantitative patterns rather than direct participant quotations. Future studies should incorporate semi-structured interviews or focus groups to capture richer narratives and validate these preliminary observations. Sixth, the study was conducted in a single city (Unaizah) within Qassim, so findings may not be transferable to other regions of Saudi Arabia, particularly rural areas where internet connectivity and digital literacy may be lower. Seventh, several interpretations in our discussion—particularly regarding cultural explanations for low privacy concern, trust in government platforms, and preferences for face-to-face care—are speculative and derived from the external literature rather than directly measured in this study. We did not specifically measure trust in government platforms, knowledge of privacy risks, cultural attitudes toward data sharing, clinician endorsement of digital health tools, costs of wearables or CGM, technical crashes or responsiveness within this cohort, or preference for face-to-face versus virtual care. These constructs should be directly measured in future research to test the hypotheses we have proposed. Eighth, we did not perform multivariable regression, path analysis, or structural equation modelling to test independent associations, mediation, or model fit. Given the low reliability of several scales, we considered such modelling premature. Future research with validated instruments should employ these advanced analytical approaches to more rigorously test the extended TAM.

### 4.4. Practical Applications

Despite these limitations, our findings have several practical implications for clinicians, health system administrators, and policymakers in Saudi Arabia.

1. For clinicians:

The strong correlation between perceived usefulness and behavioural intention suggests that healthcare providers should actively demonstrate the clinical benefits of digital health tools during consultations. For example, showing patients how a medication reminder app can reduce missed doses, or how glucose tracking data can be shared with the care team, may increase adoption. Trust was also a significant correlate; therefore, clinicians should recommend only trusted, government-endorsed platforms (e.g., Sehhaty) and explain how patient data are protected.

2. For health system administrators:

The high usage of blood glucose tracking and medication reminder apps indicates that these tools have become part of routine self-management. Administrators should ensure that these apps are interoperable with electronic health records so that data can flow seamlessly into clinical decision making. The very low uptake of telemedicine and patient portals, despite high smartphone ownership, signals a need for targeted promotional campaigns and user friendly interface improvements. As Alzghaibi [10] noted, technical barriers (crashes, slow responsiveness) and usability issues must be addressed before patients will fully embrace more advanced digital health services.

3. For policymakers:

The low privacy concern observed in this study should not be misinterpreted as a green light to neglect data security. Instead, policymakers should maintain robust data protection regulations and transparently communicate these safeguards to maintain the trust that patients currently have. However, we emphasize that our study did not directly measure trust in government platforms or knowledge of privacy risks; the low privacy concern we observed may reflect limited awareness rather than genuine confidence. Future research should distinguish between these possibilities. As highlighted by recent research, the rapid growth of IoT-generated medical data necessitates advanced privacy protections, with blockchain technology emerging as a promising solution for secure and transparent healthcare data sharing [32]. The Vision 2030 digital health transformation agenda provides an ideal framework to scale up successful interventions while continuously monitoring adoption patterns across different demographic groups. Additionally, given that self-efficacy was significantly associated with behavioral intention, policymakers should invest in digital literacy programmes, particularly for older adults and those with lower educational attainment, to ensure equitable access.

4. For technology developers:

The qualitative themes highlighted the importance of clinical utility and ease of navigation. Developers should prioritise features that directly support diabetes self-management (e.g., integration with glucometers, medication schedulers, and dietary logs) while keeping the user interface simple and intuitive. The low uptake of wearable devices (1.9%) suggests that either cost or perceived usefulness is a barrier; developers should explore more affordable options and better demonstrate the added value of wearables over smartphone only apps.

### 4.5. Future Research Directions

Future research should address the limitations of this study by using probability sampling to obtain a more representative sample, employing longitudinal designs to establish causal relationships, and using validated instruments with confirmatory factor analysis to ensure measurement reliability. Qualitative studies using in depth interviews would provide richer insights into the lived experiences of patients, particularly those who are older, less educated, or living in rural areas. Comparative studies across different regions of Saudi Arabia (e.g., Eastern Province vs. Western Province) could identify regional variations in digital health adoption. Finally, intervention studies that test the effect of clinician endorsement, digital literacy training, or technical improvements on adoption rates would provide actionable evidence for implementation.

## 5. Conclusions

This cross-sectional study with supplementary qualitative content analysis demonstrates that patients with diabetes in Qassim Unaizah, Saudi Arabia have widely adopted digital health technologies, particularly blood glucose tracking and medication reminder applications. The extended Technology Acceptance Model, incorporating perceived usefulness, attitude, self-efficacy, trust, ease of use, and privacy concerns, identified key factors associated with behavioural intention. Perceived usefulness and attitude showed the strongest associations with intention, while privacy concerns were unexpectedly low, reflecting high trust in government managed platforms and possibly cultural differences in attitudes toward data sharing. Despite high adoption of basic tools, the use of telemedicine, patient portals, and CGM remains low. However, this should not be interpreted as an inherent failure; CGM use is clinically indicated for specific patient populations, and telemedicine appropriateness depends on clinical context, patient preference, and access to infrastructure. The key opportunity lies in ensuring that these advanced tools are accessible to those who could benefit most, by addressing barriers related to awareness, cost, reimbursement, technical usability, and clinical integration, while respecting patient choice and clinical appropriateness. Clinicians, administrators, and policymakers should focus on demonstrating clinical benefits, ensuring ease of use, building trust, and addressing technical barriers to unlock the full potential of digital health in diabetes care. However, these recommendations are informed by associations observed in this cohort and should be tested in future intervention studies. The speculative explanations we have proposed—particularly regarding cultural attitudes, institutional trust, and preferences for care modality—require direct measurement in future research before they can inform policy or practice. While our findings suggest that digital health tools may have broad appeal across demographic subgroups in this population, we emphasize that this conclusion requires confirmation in larger, more representative samples. Targeted efforts remain important for ensuring equitable access, particularly for older adults, those with lower digital literacy, and populations outside urban or semi-urban areas. Future research should validate the measurement model, explore causal pathways, and develop targeted interventions to promote equitable access across all segments of the diabetic population in Saudi Arabia.

## Figures and Tables

**Table 1:** Sociodemographic characteristics of study participants (N = 203).

Characteristic	Category	n (%)
Gender	Male	127 (62.6%)
	Female	76 (37.4%)
Age (years)	Mean ± SD	47.2 ± 13.1
	Range	14–82
Education	Primary school or less	7 (3.4%)
	Intermediate school	12 (5.9%)
	Secondary school	28 (13.8%)
	High school (Diploma/vocational)	42 (20.7%)
	Bachelor’s degree	113 (55.7%)
	Master/PhD	1 (0.5%)
Employment	Full-time employed	119 (58.6%)
	Employed part-time	7 (3.4%)
	Self-employed	15 (7.4%)
	Unemployed	10 (4.9%)
	Retired	41 (20.2%)
	Student	11 (5.4%)
Income (SAR/mo)	<5000	29 (14.3%)
	5000–9999	59 (29.1%)
	10,000–14,999	63 (31.0%)
	15,000–19,999	27 (13.3%)
	≥20,000	16 (7.9%)
	Prefer not to answer	9 (4.4%)
Marital status	Single	52 (25.6%)
	Married	125 (61.6%)
	Divorced	22 (10.8%)
	Widowed	4 (2.0%)

**Table 2:** TAM construct descriptive statistics and internal consistency (N = 203).

Construct	Items (n)	Mean (SD)	Cronbach’s α	Interpretation
Perceived Usefulness (PU)	3	4.29 (0.39)	0.378	High usefulness
Ease of Use (PEOU)	4	4.11 (0.66)	0.839	High ease
Attitude (ATT)	3	4.17 (0.42)	0.354	Positive attitude
Behavioral Intention (BI)	2	4.16 (0.44)	0.498	Strong intention
Trust (TR)	2	4.22 (0.51)	0.653	High trust
Privacy Concern (PC) ^a^	3	1.70 (0.89)	0.921	High concern
Self-Efficacy (SE)	3	3.86 (0.53)	0.316	Moderate–high

^a^ Reverse-coded: lower score = higher concern.

**Table 3:** Pearson correlations between TAM constructs and BI (N = 203).

Construct	r with BI	*p* Value	Interpretation
Attitude (ATT)	0.450	<0.001	Moderate–strong
Perceived Usefulness (PU)	0.438	<0.001	Moderate–strong
Self-Efficacy (SE)	0.394	<0.001	Moderate
Trust (TR)	0.379	<0.001	Moderate
Perceived Ease of Use (PEOU)	0.340	<0.001	Moderate
Privacy Concern (PC)	0.309	<0.001	Weak–moderate

## Data Availability

The data presented in this study are available on request from the corresponding author. The data are not publicly available due to privacy or ethical restrictions.

## Abbreviations

The following abbreviations are used in this manuscript:

TAM	Technology Acceptance Model
PU	Perceived Usefulness
PEOU	Perceived Ease of Use
ATT	Attitude toward Use
BI	Behavioural Intention
TR	Trust
PC	Privacy Concern
SE	Self-Efficacy
